# Hydroxychloroquine modulates immunological pathways activated by RNA:DNA hybrids in Aicardi–Goutières syndrome patients carrying *RNASEH2* mutations

**DOI:** 10.1038/s41423-021-00657-0

**Published:** 2021-03-11

**Authors:** Jessica Garau, Daisy Sproviero, Francesca Dragoni, Elisa Piscianz, Carolina Santonicola, Davide Tonduti, Stephana Carelli, Alessandra Tesser, Gian Vincenzo Zuccotti, Alberto Tommasini, Simona Orcesi, Orietta Pansarasa, Cristina Cereda

**Affiliations:** 1 Genomic and post-Genomic Unit, IRCCS Mondino Foundation, Pavia, Italy; 2 Department of Biology and Biotechnology “L. Spallanzani”, University of Pavia, Pavia, Italy; 3 Department of Medicine, Surgery and Health Sciences, University of Trieste, Trieste, Italy; 4 Child Neurology Unit - COALA (Center for diagnosis and treatment of leukodystrophies) - Children’s Hospital “V. Buzzi”, Milan, Italy; 5 Department of Biomedical and Clinical Sciences “L. Sacco”, University of Milan, Milan, Italy; 6 Pediatric Clinical Research Center Fondazione Romeo ed Enrica Invernizzi, University of Milan, Milan, Italy; 7 Department of Pediatrics, Institute for Maternal and Child Health, “IRCCS Burlo Garofolo”, Trieste, Italy; 8 Department of Pediatrics, Children’s Hospital “V. Buzzi”, Milan, Italy; 9 Unit of Child Neurology and Psychiatry, IRCCS Mondino Foundation, Pavia, Italy; 10 Department of Brain and Behavioral Sciences, University of Pavia, Pavia, Italy

**Keywords:** Immunology, Cell biology

Aicardi–Goutières syndrome (AGS) is a rare genetic disease caused by mutations in nine genes that are all involved in nucleic acid metabolism or sensing.^1,2^ The three *RNASEH2* subunits represent the most frequently mutated genes in AGS patients,^1,3^ and mutations in *RNASEH2* subunits lead to the accumulation of endogenous RNA:DNA hybrids that may trigger an interferon-α-mediated immune response^4^ through the activation of pattern recognition receptors (PRRs).^5^ PRRs perform surveillance on extracellular, endosomal, and cytosolic compartments to identify signs of infection: endogenous nucleic acids that are inappropriately cleared may enter and accumulate in the cytoplasm, driving inflammation and autoimmune diseases.^6^ This accumulation may be the cause of the clinical autoimmune phenotype of AGS patients carrying *RNASEH2* mutations. A proven effective cure for AGS has not been discovered, and targeting these two pathways may lead to a treatment that improves patients’ immunological symptoms. Hydroxychloroquine (HCQ) interferes with normal antigen processing and presentation and is widely used in the clinical treatment of autoimmune diseases such as systemic lupus erythematosus and rheumatoid arthritis.^7^ HCQ is also a well-known inhibitor of autophagy that prevents the degradation of autolysosomes. This drug inhibits acidification and maturation of endosomes and increases the pH in lysosomes, resulting in the inhibition of their main functions, such as downstream cell signaling through TLRs.^7^ Therefore, the aim of our work was to investigate whether HCQ is able to modulate the abnormal inflammatory response driven by RNA:DNA hybrids.

Considering the noteworthy difficulties in obtaining primary cells from children affected by this rare disease, we used immortalized lymphoblastoid cell lines (LCLs): one from a patient carrying the *RNASEH2A* p.R108W + p.F230L mutations, one from a patient carrying the *RNASEH2B* p.A177T mutation and one from a healthy control. We investigated the presence of RNA:DNA hybrids using the S9.6 antibody and flow cytometry. Quantification of the mean S9.6 fluorescence revealed a significant increase in RNA:DNA hybrids only in the *RNASEH2B*-mutant LCL (Fig. S1A). RNA:DNA hybrids were primarily localized in the cytoplasm in all three cell lines, while in the control and *RNASEH2A*-mutant LCLs, RNA:DNA hybrids were distributed homogeneously; in the *RNASEH2B*-mutant LCL, they accumulated in a specific region of the cytoplasm (Fig. S1B, white arrow). These results were confirmed by immunogold staining of the RNA:DNA hybrids (Fig. S1C).

The S9.6 antibody may also bind double-stranded RNA but with a lower affinity than with RNA:DNA hybrids, and therefore, to verify the cytoplasmic accumulation of the hybrids in these cell lines, we treated the cells with RNase H.^8^ After RNase H treatment, the RNA:DNA hybrid fluorescence signal considerably decreased in the *RNASEH2B*-mutant LCL (Fig. S1D), the only cell line that presented significant accumulation of RNA:DNA hybrid. We also decided to investigate the subcellular localization of RNA:DNA hybrids by staining cells with monoclonal S9.6 antibody and specific vital dyes. While we did not find any colocalization between RNA:DNA hybrids and mitochondria or the endoplasmic reticulum in either mutant cell line, we observed colocalization of hybrids in the lysosomes in the *RNASEH2B*-mutant LCL (Fig. S1E).

We then evaluated how the RNA:DNA hybrid level changed in the healthy control LCL and the *RNASEH2B-* and *RNASEH2A-*mutant LCLs after treatment with 25 µM HCQ for 24 h. We found that in the treated *RNASEH2B*-mutant LCL, the RNA:DNA hybrid level decreased (Fig. 1A), and a loss of colocalization between lysosomes and RNA:DNA hybrids was observed (Fig. 1B). Since LysoTracker may stain both lysosomes and endosomes, we stained cells with Rab5, an early endosome marker, to clarify the RNA:DNA hybrid localization. The *RNASEH2B*-mutant LCL showed no colocalization of S9.6 and Rab5, confirming that, at the basal level, hybrids were internalized by lysosomes, whereas we discovered weak colocalization in the untreated and treated *RNASEH2A*-mutant LCL. Interestingly, Rigby et al.^9^ described the accumulation of RNA:DNA hybrids in endosomes in the presence of retroviral infection in B3T3 fibroblasts. After treatment with HCQ, we observed colocalization between Rab5 and RNA:DNA hybrids in the *RNASEH2B*-mutant LCL, highlighting a possible role of endosomes in RNA:DNA hybrid elimination. No colocalization between Rab5 and RNA:DNA hybrids was observed in the control LCL (Fig. 1C). AGS mimics congenital viral brain infections; therefore, endosomal localization, which was not evident in the control LCL, might suggest a physiological way to eliminate dangerous nucleic acids, which was not observed in the *RNASEH2B*-mutant LCL.

**Fig. 1:**
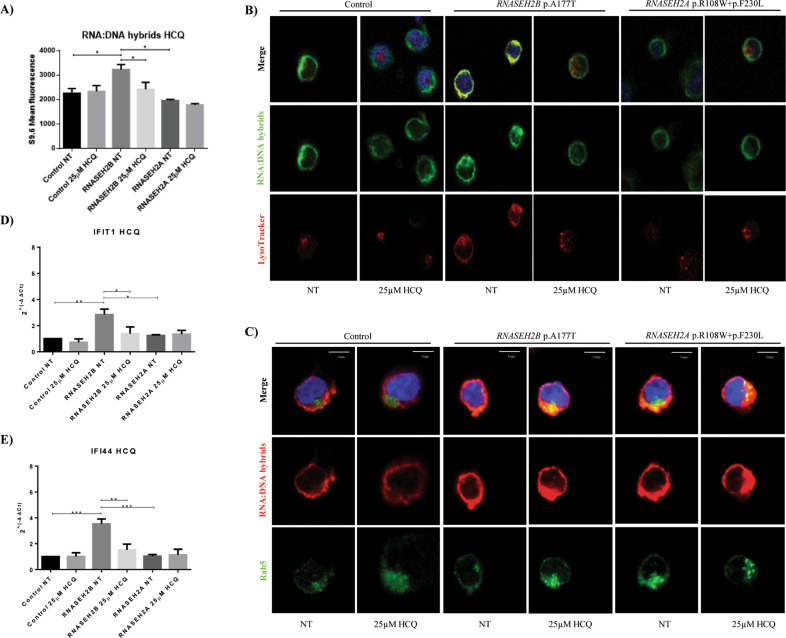
HCQ reduces and changes the localization of RNA:DNA hybrids, inhibiting the activation of interferon-stimulated genes (ISGs). **A** Accumulation of cytosolic RNA:DNA hybrids in the *RNASEH2B*-mutant LCL compared to the healthy control and *RNASEH2A*-mutant LCLs was evaluated by flow cytometry with 25 μM HCQ and without (NT) HCQ treatment. The data are presented as the means ± SEM, and significance was determined by paired *t*-test. *P < 0.05. **B** Immunofluorescence of the LCL derived from the healthy control and *RNASEH2A-* and *RNASEH2B*-mutant LCLs before (NT) and 24 h after HCQ treatment (25 μM). RNA:DNA hybrids stained with S9.6-specific mouse monoclonal antibody (green), lysosomes were stained with the endolysosomal marker LysoTracker^TM^ (red), and nuclei were stained with DAPI (blue). **C** Immunofluorescence of the LCLs derived from healthy controls and AGS patients with mutations in the *RNASEH2B* and *RNASEH2A* genes before (NT) and 24 h after HCQ treatment (25 μM). RNA:DNA hybrids were stained with S9.6-specific mouse monoclonal antibody (red), endosomes were stained with Rab5 (green), and nuclei were stained with DAPI (blue). **D**, **E** mRNA expression levels of *IFIT1* and *IFI44*, two ISGs, in healthy controls and AGS patients before and after HCQ treatment. The data are presented as the means ± SEM, and significance was determined by ANOVA and Tukey’s post hoc test. *P < 0.05; **P < 0.01; ***P < 0.001

Since HCQ also interferes with autophagy,^7^ we investigated whether RNA:DNA hybrid is depleted upon HCQ treatment via the autophagy pathway. After treating the cells with HCQ, we observed an increase in the LC3-II/LC3-I ratio for each cell line, especially in the *RNASEH2B*-mutant LCL, with a significant *P* value < 0.001 (Fig. S2A). Moreover, LC3 colocalized with RNA:DNA hybrids in *RNASEH2B*-mutant LCL after HCQ treatment (Fig. S2C). In accordance with these results, we observed increased p62 protein levels after HCQ treatment and p62 colocalization with RNA:DNA hybrids in the *RNASEH2B-*mutant LCL, which became more evident after HCQ treatment (Fig. S2B, D), strengthening the possible role of autophagy in RNA:DNA hybrid discards.

After HCQ treatment, cGAS protein levels slightly decreased in the *RNASEH2B*-mutant LCL (Fig. S3A), and subsequently, the same behavior was described for IRF3 transcript levels. We also found decreased MYD88 and IRF7 transcript levels, whereas no difference in NF-kB transcripts was evident in the AGS LCLs (Fig. S3C). To confirm these results, we used flow cytometry to evaluate the phosphorylation of TBK1, which links the recognition of nucleic acids to the development of a type I IFN response through cGAS activation.^10^ No significant differences in the phosphorylation profile were identified before or after treatment with HCQ (Fig. S3B). We also evaluated how the expression of two ISGs, *IFI44* and *IFIT1*, changed in *RNASEH2A-* and *RNASEH2B-*mutant LCLs after treatment. *IFI44* and *IFIT1* expression levels decreased after HCQ treatment in the *RNASEH2B*-mutant LCL, with a greater decrease in *IFIT1*, whereas no differences in the *RNASEH2A*-mutant LCL were observed (Fig. 1D, E).

In conclusion, we found that RNA:DNA hybrids accumulate mainly in the cytoplasm and colocalize with lysosomes in the LCL derived from an AGS patient carrying a *RNASEH2B* mutation, which may indicate an impairment in the RNA:DNA hybrid degradation process. On the other hand, the *RNASEH2A*-mutant LCL carries RNA:DNA hybrids in endosomes, possibly explaining the lack of impaired RNA:DNA hybrid degradation in this cell line. Moreover, we found that HCQ is a drug that can induce decreased activation of the IFN-α immune cascade only in the *RNASEH2B*-mutant LCL, the only cell line presenting high ISGs. Therefore, we hypothesized that HCQ, a European Medicines Agency-approved drug, may be an effective treatment for AGS patients who present abnormal activation of the innate immune response.

## Supplementary information

Materials and Methods

Figure S1

Figure S2

Figure S3
