# Supplementary material for: Hydroxychloroquine modulates immunological pathways activated by RNA:DNA hybrids in Aicardi–Goutières syndrome patients carrying RNASEH2 mutations
Source: Cell Mol Immunol. 2021 Mar 11;18(6):1593–5. doi: 10.1038/s41423-021-00657-0 (PMC8166873; doi:10.1038/s41423-021-00657-0)
Supplement: Supplementary file 1 — Materials and Methods [file 41423_2021_657_MOESM1_ESM.docx]

Materials and Methods

*Patients’ enrollment*

Patients affected by AGS were enrolled at the IRCCS Mondino Foundation, Pavia, Italy. The two patients mutated in *RNASEH2B* (p.A177T) and *RNASEH2A* (p.R108W + p.F230L) were diagnosed according to clinical suggestion guided by defined criteria^11,12^ and the genetic analysis has been performed as reported in Garau et al., 2019^3^. The healthy volunteer, free from any pharmacological treatment and pathology, was recruited at the Transfusion Centre of the IRCCS Policlinico S. Matteo Foundation in Pavia, Italy. All samples were obtained with informed consent.

*Ethical statement*

All procedures performed in this study involving human participants were in accordance with the ethical standards of the institutional and/or national research committee and with the 1964 Helsinki declaration and its later amendments or comparable ethical standards. The study design was examined by the IRBs of the enrolling Institutions (Protocol n°375/04 of 07/01/2004; n° 3549/2009 of 30/9/2009 and 11/12/2009, and n°20170035275 of 23/10/2017).

*Cells isolation*

Peripheral blood mononuclear cells (PBMCs) were immediately isolated from peripheral venous blood using Histopaque®-1077 (Sigma-Aldrich, St. Louis, MO, USA), following manufacturer’s specifications. Briefly, blood samples were carefully posed in a centrifuge tube over an equal volume of Histopaque®-1077 and centrifuged at 1800 RPM for 30 minutes with low deceleration. PBMCs were then recovered from the intermediate phase and washed with 1X PBS (Sigma-Aldrich, St. Louis, MO, USA). Collected cells were centrifuged at 1600 RPM for 10 minutes and supernatant was discarded.

*LCLs culture*

LCLs (EBV-immortalized at Laboratorio di Genetica Umana, IRCCS Istituto Giannina Gaslini, Genoa) carrying mutations in *RNASEH2A* (p.R108W + p.F230L) and *RNASEH2B* (p.A177T) genes and one healthy control were analyzed. LCLs were maintained in RPMI 1640 medium (CARLO ERBA Reagents S.r.l., Cornaredo, Italy), supplemented with 20% fetal bovine serum (FBS) (CARLO ERBA Reagents S.r.l., Cornaredo, Italy), 0.3 mg/L L-glutamine and 5% penicillin-streptomycin (CARLO ERBA Reagents S.r.l., Cornaredo, Italy), at 37 °C in a humidified atmosphere with 5% of CO2. At need, cells were pelleted by centrifugation, washed with 1X PBS and further processed as required. LCLs of healthy controls and mutated patients were treated with 25 µM of HCQ (Sigma-Aldrich, St. Louis, MO, USA) for 24 h according to the literature^13^.

*Immuno-gold labelling of anti-RNA:DNA antibody*

Approximately 3×10^6^ cells were washed in 1X PBS (Sigma-Aldrich, St. Louis, MO, USA) and incubated with the fixing solution (2.5% glutaraldehyde and 2% paraformaldehyde in cacodylate buffer, pH 7.3) at 4°C for 4 hours, followed by a post-fixation step in 1.5% osmium tetroxide for 1 hour at room temperature (RT) and Epon-Araldite embedding^14^. Ultra-thin Lowicryl sections were mounted on formvar nickel grids and washed in 5% BSA (1X PBS, 0.05% Triton X-100) for 10 minutes. Then the grids were incubated with the anti-RNA:DNA hybrid antibody (MABE1095, Sigma-Aldrich, St. Louis, MO, USA) diluted 1:300 in 1X PBS for 1 hour at RT. After washing in 1X PBS, they were floated in protein A conjugated to 10 nm colloidal gold particles (Sigma-Aldrich, St. Louis, MO, USA), diluted 1:25 in 1X PBS for 45 minutes at RT. They were washed in 1X PBS and then in bidistilled water. Finally, the sections were counterstained with 5% uranyl acetate and observed in a Hitachi EM H-600-2 at 75 kV^15^.

*RIPA proteins extraction and quantification*

Soluble protein samples were obtained with the extraction using RIPA buffer (50mM Tris-HCl [pH 8.0], 150mM NaCl, 1% NP-40, 12mM Deoxycolic acid, supplemented with protease inhibitors). Protein concentration was determined using bicinchoninic acid (BCA) method (Sigma-Aldrich, St. Louis, MO, USA) and bovine serum albumin (BSA) (Sigma-Aldrich, St. Louis, MO, USA) as standard. Proteins quantification was determined using NanoDrop (Thermo Fisher Scientific, Waltham, MA, USA).

*Western blot*

Western Blotting analysis was performed by SDS–polyacrylamide gel electrophoresis (SDS-PAGE). Samples containing 30μg of proteins were loaded into 12.5% SDS-PAGE gel. Then, samples were transferred to PVDF membranes using a semidry transfer apparatus (Trans-blot Turbo, Bio-Rad Laboratories, Hercules, CA, USA). Membranes were treated with a blocking solution, containing 5% of non-fat dry milk in 1X TBS-T buffer (10mM Tris-HCl, 100mM NaCl, 0.1% Tween, pH 7.5), for 1h and then incubated overnight with the primary antibodies at 4°C. Immunoreactivity was detected using the donkey anti-rabbit or anti-mouse secondary peroxidase-conjugated (GE Healthcare, UK). The immunoreactive bands were visualized using the enhanced chemiluminescence detection kit (ECL Advance, GeHealthcare, UK). The following antibodies were used for western blot analysis: mouse monoclonal anti-cGAS (sc-515777, Santa Cruz Biotechnology, Dallas, TX, USA, dilution 1:500), rabbit polyclonal anti-LC3 (L8918, Sigma-Aldrich, St. Louis, MO, USA, dilution 1:500), rabbit polyclonal anti-SQSTM1/p62 (ab91526, Abcam, Cambridge, UK, dilution 1:500). Rabbit polyclonal anti-GAPDH (GTX100118, GeneTex, Irvine, CA, USA, dilution 1:10000) has been used as loading control.

*Immunofluorescence Confocal Microscope*

About 1x10^5^ cells were placed on a poly-L-Lysine slide (Thermo Fisher Scientific, Waltham, MA, USA) and incubated at RT for 20 minutes. Cells were stained with ER-Tracker™ (1µM) or Mito-Tracker™ (100nM) red dyes (Thermo Fisher Scientific Inc., Waltham, MA, USA) and then incubated for 30 minutes at 37°C. Cells were also stained with endolysosomal marker LysoTrackerTM (75 nM) red dye (Thermo Fisher Scientific Inc., Waltham, MA, USA) diluted in 1X PBS and then incubated for 1 hour and 30 minutes at 37°C. Cells were then fixed using a solution of 4% paraformaldehyde for 15 minutes at RT. Fixed cells were then permeabilized with 0.1% Triton™ X-100 (Sigma-Aldrich, St. Louis, MO, USA) for 10 minutes. Samples were treated with a blocking solution (0,05% Triton™ X-100, 1% BSA in 1X PBS) for 1 hour and were incubated with the primary antibody overnight at 4°C and incubated with secondary antibody for 1 hour at RT. They were finally washed 1X PBS, mounted with ProLong® Gold Antifade Reagent with DAPI (Thermo Fisher Scientific, Waltham, MA, USA), dried and nail-polished. Slides were analyzed with a confocal laser microscope using z-stack acquisition (Leica DM IRBE, Leica Microsystems Srl, Italy). The following antibodies were used for IF: mouse monoclonal anti-S9.6 (MABE1095, Sigma-Aldrich, St. Louis, MO, USA, dilution 1:250), rabbit monoclonal anti-Rab5 (C8B1) (#3547S, Cell Signaling Technology, Danvers, MA, USA, dilution 1:250), rabbit polyclonal anti-LC3 (L8918, Sigma-Aldrich, St. Louis, MO, USA, dilution 1:250), rabbit polyclonal anti-SQSTM1/p62 (ab91526, Abcam, Cambridge, UK, dilution 1:250). CFTM 594 goat anti-mouse (Sigma-Aldrich, St. Louis, MO, USA, dilution 1:750) and CFTM 488A goat anti-rabbit (Sigma-Aldrich, St. Louis, MO, USA, dilution 1:750) were used as secondary antibodies

*RNA extraction*

RNA from LCLs was extracted with Trizol® reagent (Sigma-Aldrich, St. Louis, MO, USA) according to manufacturer’s specifications. RNA was then quantified by NanoDrop ND1000 UV-Vis Spectrophotometer (Thermo Fisher Scientific, Waltham, MA, USA).

*Reverse transcription and Real-Time PCR*

RNA (800 ng) was reverse transcribed using the iScript™ Reverse Transcription Supermix kit for RT-qPCR (Bio-Rad Laboratories, Hercules, CA, USA), according to the manufacturer’s recommendations. The relative abundance of target transcripts was measured using TaqMan probes (Applied Biosystems, Foster City, CA, USA) for *IFI44L* and *IFIT1* (Hs00199115_m1 and Hs00356631_g1) and normalized to the expression level of *HPRT1* (Hs03929096_g1) as described in literature^16^. To perform this assay, the CFX96™ Real-Time PCR Detection System Bio-Rad Laboratories, Hercules, CA, USA) has been used.

For *MYD88*, *NF-kB*, *IRF3* and *IRF7* analysis qPCR reactions included 200 nM of each oligonucleotide, 1 μL of SYBR Green SuperMix (BioRad, Richmond, CA, USA), and 1 μL of cDNA template (or water control). Primers are indicated in Table 1. Cycle threshold (Ct) values were automatically recorded for each replicate qPCR reaction, and mean Ct values were normalized against those determined for GAPDH. Fold-expression differences relative to healthy controls were determined using the 2^-ΔΔCt^ method.

*Flow cytometry*

About 1x10^6^ cells were collected for each condition. For viability staining, Zombie Violet dye solution (BioLegend, San Diego, CA, USA) (1:500 in 1X PBS) was used. Cells were then labelled with anti-CD19 APC-H7 or isotype control antibody (BD Biosciences, Franklin Lakes, NJ, USA). Both tubes were centrifuged at 1600 RPM for 5 minutes, the supernatant was removed and a permeabilizing factor (BD Cytofix/Cytoperm™ Fixation and Permeabilization Solution, BD Biosciences, Franklin Lakes, NJ, USA) was added at RT. Cells for each tube were rinsed with a washing buffer (1X PBS, perm/wash 1X, 2% FBS) and centrifuged twice at 1600 RPM for 5 minutes. The resulting pellets were resuspended with the perm/wash solution and labelled with anti-S9.6 PE and isotype control for 1h at RT. S9.6 labelled with PE has been obtained with PE/R-Phycoerythrin Conjugation Kit (Abcam, Cambridge, UK). Samples were analysed immediately after labelling, using a BD FACS Canto II with BD FACS Diva software (BD Biosciences, Franklin Lakes, NJ, USA).

Phosphorylated-TBK1 was measured in flow cytometry by intracellular staining with specific antibodies. Briefly, cells were firstly fixed with PBS without Ca/Mg (CARLO ERBA Reagents S.r.l., Cornaredo, Italy) with 4% Paraformaldehyde for 10 minutes and after treated with 500 uL of cold 90% Methanol. After washing, LCLs were stained for 1h in the dark at room temperature with anti-pTBK1 antibody (pTBK1/NAK1, ser172, clone D52C2, rabbit, PE, final concentration 4 ug/mL, Cell Signaling Technology, Danvers, MA, USA) or with the isotype control (rabbit DA1E mAb IgG XP ™ isotype control, PE, final concentration 4 ug/mL, Cell Signaling Technology, Danvers, MA, USA) and recovered to proceed with cytofluorimetric analysis on MacsQuant 10 (Miltenyi Biotec, Bergisch Gladbach, Germany). Results were processed based on pTBK1-positive cells compared to isotype control after morphologic gating to exclude debris.

*Statistical analysis*

Values were expressed as means ± S.D. Statistical analysis was performed by t-test and One-Way Analysis of Variance (ANOVA) followed by Tukey's Test as a post-hoc test (GraphPad Prism version 5, USA). Values were considered statistically significant when *P* values were < 0.05. No bars in the graph were indicated when the results were not statistical significant.

**References**

1. Orcesi, S., La Piana, R., Fazzi, E. Aicardi-Goutieres syndrome. Br. Med. Bull. **89**, 183-201 (2009).
2. Tonduti, D. et al. Encephalopathies with intracranial calcification in children: clinical and genetic characterization. Orphanet J Rare Dis. **13(1)**, 135 (2018).
3. An, J., Woodward, J. J., Sasaki, T., Minie, M. & Elkon, K. B. Cutting edge: Antimalarial drugs inhibit IFN-β production through blockade of cyclic GMP-AMP synthase-DNA interaction. J Immunol. **194(9)**, 4089-93 (2015).
4. Necchi, V., Sommi, P., Ricci, V. & Solcia, E. In vivo accumulation of Helicobacter pylori products, NOD1, ubiquitinated proteins and proteasome in a novel cytoplasmic structure. PLoS One. **5(3)**, e9716 (2010).
5. Tao, W., Xu, W., Valdivia, M. M., Hao, S. & Zhai, Z. H. Distribution and Transcription Activity of Nucleolar DNA in Higher Plant Cells. Cell Biol Int. **25(11)**, 1167-71 (2001).
6. Rice, G. I. et al. Assessment of interferon-related biomarkers in Aicardi-Goutières syndrome associated with mutations in TREX1, RNASEH2A, RNASEH2B, RNASEH2C, SAMHD1, and ADAR: a case-control study. Lancet Neurol. **12(12)**, 1159-69 (2013).

Table 1. Primers sequences

| Gene symbol | Forward sequence | Reverse sequence |
| --- | --- | --- |
| MYD88 | TGTCTGCGACTACACCAACC | ACAACGAAAGGAGGAGGCAG |
| NF-kB | ACAGCTGGATGTGTGACTGG | TCCTCCGAAGCTGGACAAAC |
| IRF3 | GAGGTGACAGCCTTCTACCG | TGCCTCACGTAGCTCATCAC |
| IRF7 | ATGGGCAAGTGCAAGGTGTA | GATGGTATAGCGTGGGGAGC |
| GAPDH | ATGGAAATCCCATCACCAT | CGCCCCACTTGATTTTGG |
