# Supplementary figures and images for: Hydroxychloroquine modulates immunological pathways activated by RNA:DNA hybrids in Aicardi–Goutières syndrome patients carrying RNASEH2 mutations
Source: Cell Mol Immunol. 2021 Mar 11;18(6):1593–5. doi: 10.1038/s41423-021-00657-0 (PMC8166873; doi:10.1038/s41423-021-00657-0)

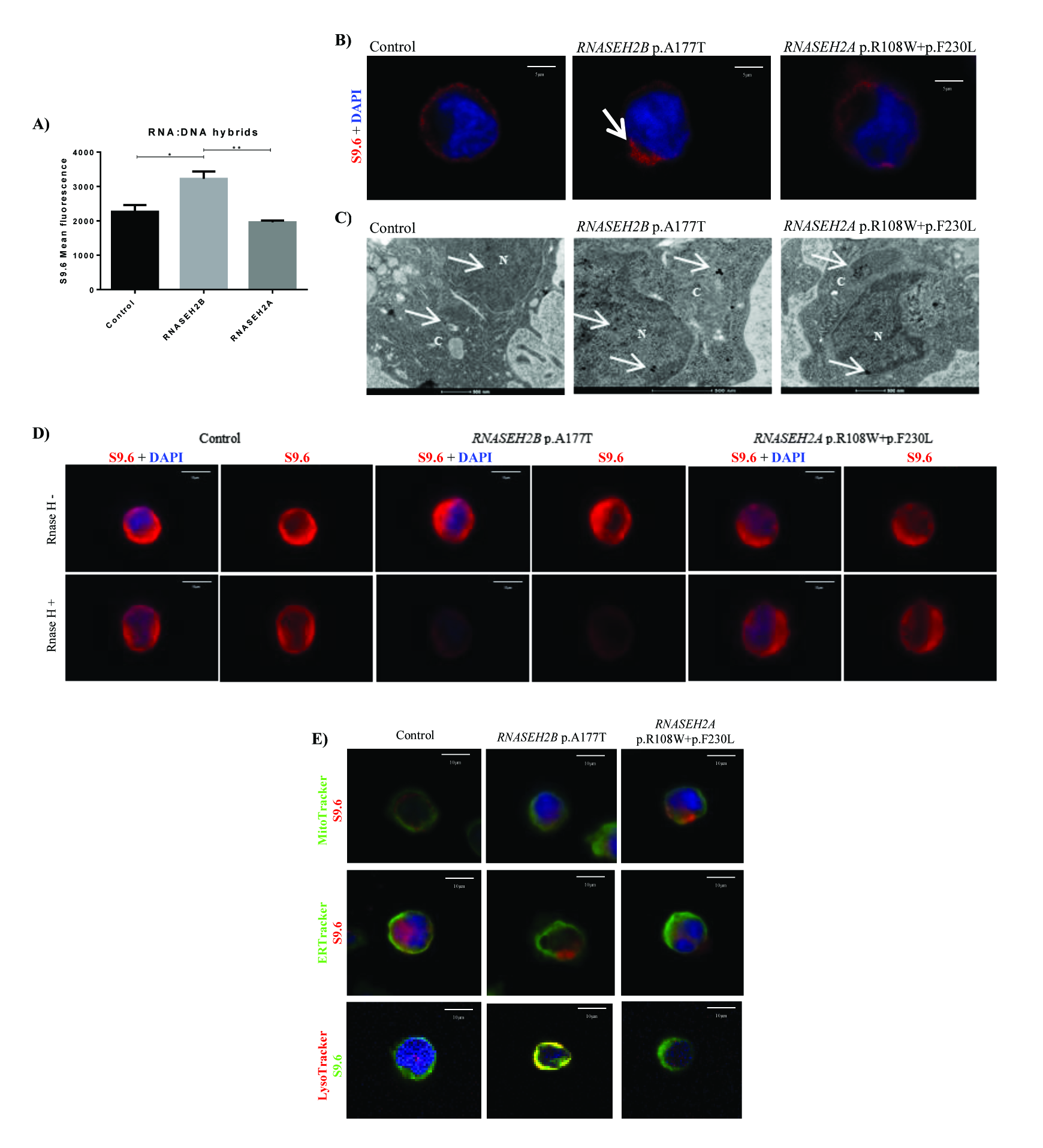

Supplement: Supplementary file 2 — Figure S1 [file 41423_2021_657_MOESM2_ESM.tif]

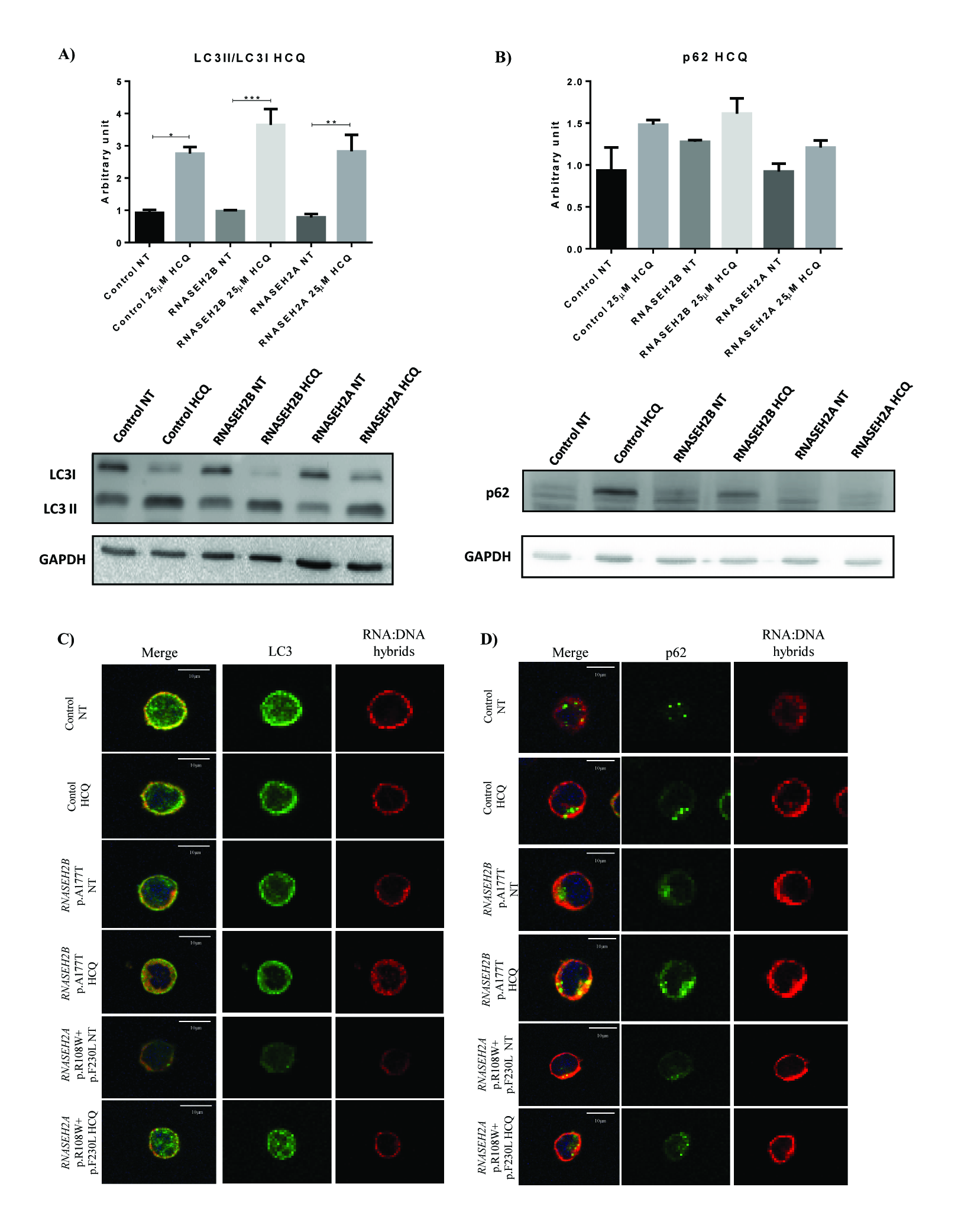

Supplement: Supplementary file 3 — Figure S2 [file 41423_2021_657_MOESM3_ESM.tif]

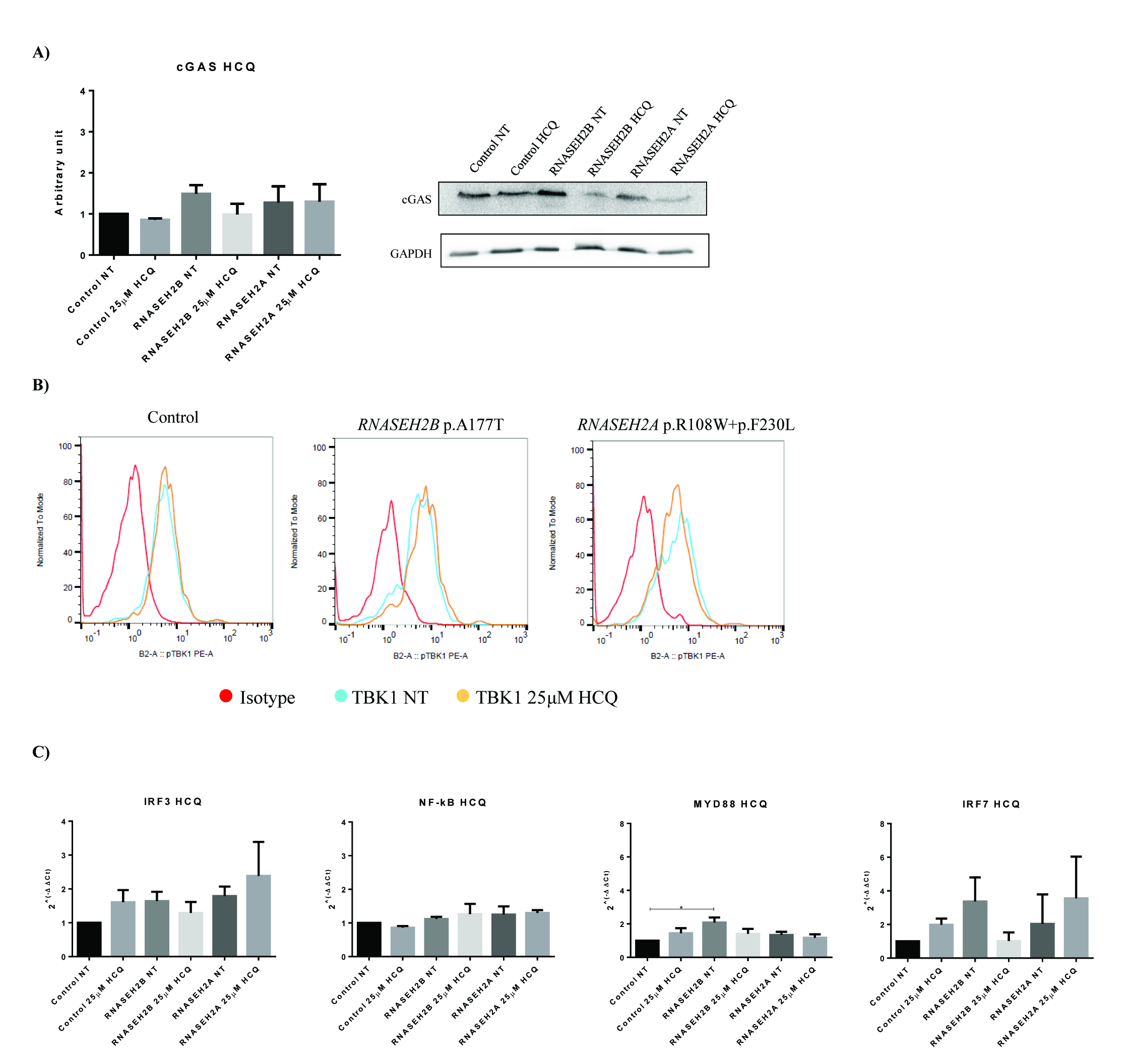

Supplement: Supplementary file 4 — Figure S3 [file 41423_2021_657_MOESM4_ESM.tif]
